# Adénocarcinome de l’ampoule de Vater révélé par un prurigo

**DOI:** 10.11604/pamj.2017.28.154.13846

**Published:** 2017-10-18

**Authors:** Ilhame Naciri, Baderddine Hassam

**Affiliations:** 1 Service de Dermatologie et Vénérologie, Centre Hospitalier Universitaire IBN Sina, Faculté de Médecine et de Pharmacie, Université Mohammed V, Rabat, Maroc

**Keywords:** Adénocarcinome, ampoule de Water, ampullome, prurigo, Adenocarcinoma, ampulla of Vater, ampulloma, prurigo

## Abstract

Chronic prurigo may reveal several internal diseases. Its association with a solid neoplasia is rare. We report an exceptional case of chronic prurigo revealing surgically curable adenocarcinoma of the ampulla of Vater. Our study involved a 70-year old patient, with no particular previous history, who was hospitalized for etiological assessment of prurigo lasting for 06 months associated with non-documented weight loss. The lesions were isolated and involved the trunk and the limbs (A); initial assessment was strictly normal. Given the lack of patient’s improvement with symptomatic treatment, additional exams were performed, including thoracoabdominopelvic CT scan, which objectified a tumor of the biliopancreatic junction measuring 2 cm, suggesting the diagnosis of ampulloma (B). The patient underwent surgical excision of this adenocarcinoma of the ampulla of Vater followed by regression of the skin lesions without further treatment. At 1-year follow-up, there was no evidence of recurrence of the skin lesions and the patient achieved complete remission of adenocarcinoma.

## Image en médecine

Le prurigo chronique peut être révélateur de plusieurs maladies internes. L’association à une néoplasie solide est rare. Nous rapportons un cas exceptionnel d’un prurigo chronique révélateur d’un adénocarcinome de l’ampoule de Vater, curable chirurgicalement. Il s’agit d’un homme âgé de 70 ans, sans antécédent particulier, qui était hospitalisé pour bilan étiologique de son prurigo évoluant depuis 06 mois dans un contexte d’amaigrissement non chiffré. Les lésions étaient isolées intéressaient le tronc et les membres (A), le bilan initialement réalisé était strictement normal. Devant l’absence d’amélioration sous traitement symptomatique, un complément d’investigations comprenant notamment une tomodensitométrie thoraco-abdominopelvienne objectivait un processus tumoral du confluant bilio pancréatique de 2 cm, évoquant le diagnostic d’ampullome (B). Après un traitement chirurgical de cet adénocarcinome de l’ampoule de Vater, les lésions cutanées régressaient sans aucun traitement complémentaire. Un an plus tard, il n’existait pas de rechute des lésions cutanées et le malade était en rémission complète de son adénocarcinome.

Fig 1

**Figure 1:**
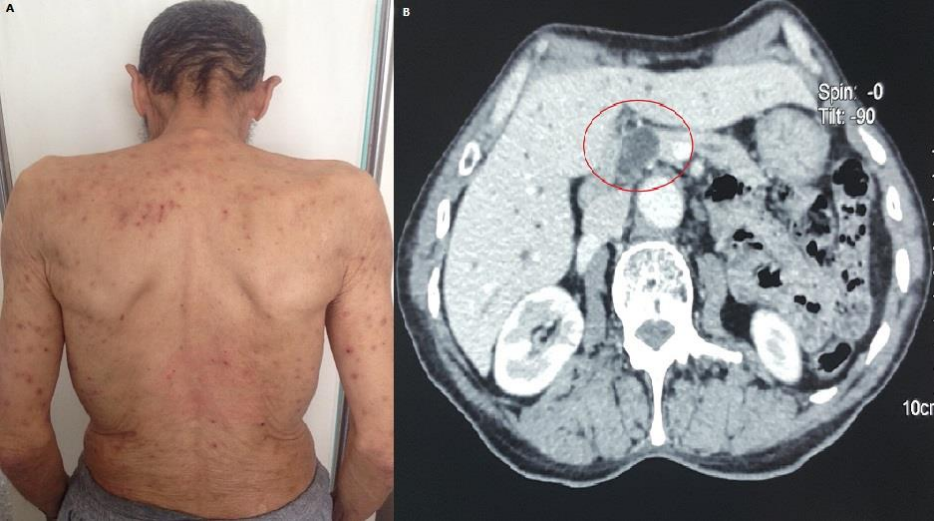
A) lésions excoriées de prurigo, sur le dos et la racine des membres supérieurs; B) tomodensitométrie thoraco-abdomino-pelvienne: processus tumoral du confluant bilio pancréatique

